# Identification of candidate flowering and sex genes in white Guinea yam (*D*. *rotundata* Poir.) by SuperSAGE transcriptome profiling

**DOI:** 10.1371/journal.pone.0216912

**Published:** 2019-09-23

**Authors:** Gezahegn Girma, Satoshi Natsume, Anna Vittoria Carluccio, Hiroki Takagi, Hideo Matsumura, Aiko Uemura, Satoru Muranaka, Hiroko Takagi, Livia Stavolone, Melaku Gedil, Charles Spillane, Ryohei Terauchi, Muluneh Tamiru

**Affiliations:** 1 Bioscience center, International Institute of Tropical Agriculture (IITA), Ibadan, Oyo State, Nigeria; 2 Plant and AgriBiosciences Research Centre (PABC), Ryan Institute, National University of Ireland Galway, Galway, Ireland; 3 Department of Genomics and Breeding, Iwate Biotechnology Research Center (IBRC), Kitakami, Iwate, Japan; 4 Japan International Research Center for Agricultural Sciences (JIRCAS), Ohwashi, Tsukuba, Japan; University of Tsukuba, JAPAN

## Abstract

Dioecy (distinct male and female individuals) and scarce to non-flowering are common features of cultivated yam (*Dioscorea* spp.). However, the molecular mechanisms underlying flowering and sex determination in *Dioscorea* are largely unknown. We conducted SuperSAGE transcriptome profiling of male, female and monoecious individuals to identify flowering and sex-related genes in white Guinea yam (*D*. *rotundata*), generating 20,236 unique tags. Of these, 13,901 were represented by a minimum of 10 tags. A total 88 tags were significantly differentially expressed in male, female and monoecious plants, of which 18 corresponded to genes previously implicated in flower development and sex determination in multiple plant species. We validated the SuperSAGE data with quantitative real-time PCR (qRT-PCR)-based analysis of the expression of three candidate genes.

We further investigated the flowering patterns of 1938 *D*. *rotundata* accessions representing diverse geographical origins over two consecutive years. Over 85% of accessions were either male or non-flowering, less than 15% were female, while monoecious plants were rare. Intensity of flowering varied between male and female plants, with the former flowering more abundantly than the latter. Candidate genes identified in this study can be targeted for further validation and to induce regular flowering in poor to non-flowering cultivars. Findings of the study provide important inputs for further studies aiming to overcome the challenge of flowering in yams and to improve efficiency of yam breeding.

## Introduction

White Guinea yam, *Dioscorea rotundata* Poir., is the most preferred and widely cultivated yam species in West-Africa [1]. Despite its considerable economic and socio-cultural importance, genetic improvement of cultivated yam remains difficult and slow due to its dioecy and poor to non-flowering nature [2]. Dioecy, the presence of distinct male and female individuals, is one of the major characteristics of the genus *Dioscorea* [3]. A major breeding challenge associated with dioecy is that synchronizing flowering time is difficult when making genetic crosses. In addition, many cultivars of *D*. *rotundata* rarely flower, while a significant proportion of those that do flower seldom set fertile seeds [4].

Mechanisms of sex determination in plants have been investigated in over 40 angiosperm species, which identified both heteromorphic and monomorphic sex chromosomes as well as XY and ZW sex-determination systems [5,6]. Due to the large number and small sizes of chromosomes, the identification of sex chromosomes at cytological level has remained difficult in *Dioscorea* [7]. In *D*. *tokoro*, a diploid wild species, and *D*. *alata* (water or winged yam), a heterogametic (XY: male) and homogametic (XX: female) sex has been proposed based on segregation patterns of AFLP markers tightly linked to sex and QTL analysis, respectively [5,6,8]. However, a more recent study has revealed that sex determination in the cultivated African species of *D*. *rotundata* follows the ZZ (male) and ZW (female) system [9]. In the same study, a candidate chromosomal region associated with sex was determined and a diagnostic marker for sex determination developed. However, gene(s) functionally responsible for sex determination are yet to be identified in yam species.

In angiosperms, flower development involves gene expression and pathway changes in vegetative meristems, leading to their conversion to flowering meristems in response to environmental cues and developmental signals [10]. Molecular genetic analyses in eudicots such as *Arabidopsis thaliana* and *Antirrhinum majus* have led to the identification of several floral organ-identity genes, which were originally grouped into three classes (class A, B, and C) based on the floral organ identity they specify [11,12]. The class A, B, and C genes are required for the development of sepals and petals, petals and stamens, and stamens and carpels, respectively. Class A and C genes are mutually antagonistic. As master regulators of floral organ identity, plant MADS-box transcription factors are central to the ABC model [13]. Further studies revealed that additional gene classes, class D and E, are important for ovule and organ development, respectively, leading to a modified ABCDE model for flower development [14].

A range of factors can regulate sex differentiation in angiosperms including genetic [15], epigenetic and environmental [16], as well as physiological regulation by phytohormones [17]. Understanding the molecular and genetic mechanisms of flowering is essential for efficient plant breeding. Recently, whole genome sequencing of *D*. *rotundata* has allowed the identification of a genomic region associated with sex [9]. This finding is expected to open opportunities for dissecting the molecular mechanisms regulating flowering and sex determination in yam. Identification of flowering and sex-related genes is critical to scaling up utilization of the available yam germplasm in breeding programs.

Various sequencing-based transcriptome profiling techniques are available for gene expression profiling, novel gene discovery, and genome annotation studies. These include Serial Analysis of Gene Expression (SAGE), which is based on unique short sequence tags 14–15 bp [18], LongSAGE which uses a different type IIS enzyme, *Mme*I, to generate 21-bp fragments from each transcript [19], Robust-LongSAGE (RL-SAGE) [20], Expressed Sequence Tag Analysis (EST) Analysis (Nielsen 2006) [20], Digital Gene Expression TAG (DGE-TAG), DeepSAGE [21], and RNA-Seq [22]. The next-generation sequencing (NGS)-based and high-throughput SuperSAGE for tag-based gene expression profiling involves sequencing of longer fragments (26-bp) and simultaneous analysis of multiple samples by using indexing (barcoding) [23]. The longer tags generated by SuperSAGE compared to the relatively shorter tag reads obtained by other NGS-based techniques such as DGE-TAG significantly improve the accuracy of tag-to-gene annotation [23].

In this study, we applied SuperSAGE to analyze transcriptome changes in flowers at early developmental stages from *D*. *rotundata* accessions representing three flowering groups (male, female and monoecious) and identified genes that are differentially expressed among these flowering groups. We show that majority of these differentially expressed genes (DEGs) correspond to genes implicated in regulating flowering and sex determination in multiple species. Our findings suggest that known mechanisms underlying flowering and sex determination are also likely to be conserved in *D*. *rotundata*.

## Materials and methods

### Morphological characterization

A total of 1938 *D*. *rotundata* accessions planted for routine field maintenance at IITA Genetic Resources Center in Ibadan (Nigeria) were characterized using 12 morphological traits for two consecutive seasons in 2010 and 2011.The flowering pattern of these accessions was also recorded over the same period. The 12 morphological traits were selected among the yam descriptors jointly developed by the former International Plant Genetic Resources Institute (current known by its new designation Bioversity International) and the International Institute of Tropical Agriculture [24]. Detailed description of the traits used is provided in Supplemental Table 1.

**Table 1:** Summary of SuperSAGE tags generated by Illumina sequencing of *D*. *rotundata* accessions representing different flowering groups.

Accession	>Sex	Total tags	Unique tags	Non- singleton tags
TDr3631	Male	1,251,361	17,773	16,602
TDr2965	Male	1,460,689	18,234	17,408
TDr4087	Female	1,049,552	18,620	17,853
TDr1679	Female	560,257	17,534	15,887
TDr4162	Monoecious	1,209,229	18,032	17,146
TDr1506	Monoecious	1,309,189	18,746	18,177
TDr1819	Monoecious	1,492,246	18,918	18,403

### Sampling and RNA extraction for SuperSAGE

**Fig 1:**
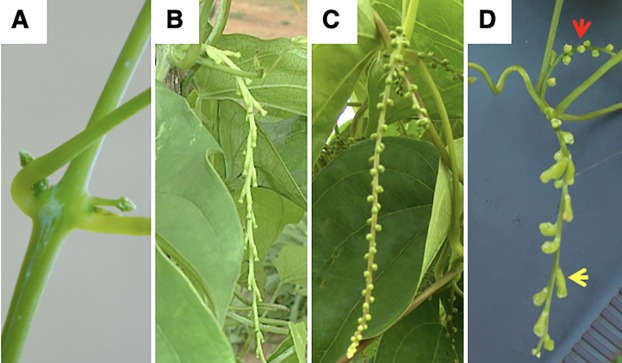
Flower types in yam (*D*. *rotundata*). **(**A) Example of flowers at early growth stage which also corresponded to the stage at which samples were collected for SuperSAGE analysis, (B) female flowers, (C) male flowers, and (D) monoecious plant with separate male (red arrow) and female (yellow arrow) flowers.

Tubers harvested from seven accessions including two male, two female, and three monoecious (Table 1) selected from the accessions used for morphological characterization based on their consistency of flowering over two years were planted in pots in a screen house. Samples for RNA extraction were collected from early stage flowers (Fig 1). Total RNA was extracted using the Qiagen RNeasy plant mini kit according to manufacturer’s protocol (Qiagen, Venlo, the Netherlands). On-column DNAase treatment was performed to remove contaminating DNA.

### SuperSAGE: Library preparation and sequencing

SuperScript II double-strand cDNA synthesis kit was used for cDNA synthesis with the biotinylated adapter-oligo (dT) primer (5’-bio-CTGATCTAGAGGTACCGGATCC-CAGCAGTTTTTTTTTTTTTTTTT-3’). Synthesized cDNA was purified using Qiagen PCR purification Kit. The library was prepared following the protocols of Matsumura et al. [23]. Briefly, the NlaIII anchoring enzyme was used for sample digestion, and the fragments (NlaIII-digested cDNA) were bound to streptavidin-coated magnetic beads (Dynabeads streptavidin M-270). Non-biotinylated cDNA fragments were removed by washing, and then adapter 2 was ligated to digested cDNA fragments bound to the magnetic beads. The type III restriction enzyme EcoP151 was used for digestion of adapter 2-cDNA after washing. Adapter2-26bp fragments were further ligated to adapter 1 (that are specific for each of the samples) as described Matsumura et al. [23] were used.

The adapter2-tag-adapter1 ligates was amplified using Phusion High polymerase and GEX primers (5’-AATGATACGGCGACCACCGACAGGTTCAGAGTTCTACAGTCCGA-3’ and 5’-CAAGCAGAAGACGGCATACGATCT-3’). The amplification program was 98°C for 1min, 10 cycles at 98°C for 35 sec, and 60°C for 30 sec. The PCR product consisting of eight tubes per sample was pooled and concentrated using Qiagen MinElute reaction purification kit. The amplification product was run on an 8% non-denaturing polyacrylamide gel. After staining with SYBR green (Takkara Bio), the DNA band around 123-125-bp size was cut out and gel purified. The purified PCR product from each sample was analyzed for its quantity and quality on an Agilent Bioanalyzer 2. The PCR product was cloned using invitrogen:—zeroblunt Topo PCR cloning kit for sequencing and later transformation using one shot chemical transformation protocol. Colony PCR was performed using 2x colony PCR mixture and purified using QIAGEN PCR purification kit. Purified and mixed PCR products were applied to Illumina Genome Analyzer II for sequencing reactions following the manufacturers protocol.

### Real-time quantitative reverse transcription PCR (qRT-PCR)

Total RNA was extracted from female and male flowers after flowering using the CTAB extraction protocol as described by Dellaporta [25] with slight modifications. Approximately 500 mg tissues were grinded using mortar and pestle in liquid nitrogen. A pre-heated 1000 uL of CTAB extraction buffer (2% CTAB, 2% PVP-40, 20 mM Tris–HCl pH 8.0, 1.4 M NaCl, 20 mM EDTA) was added to each sample and incubated at 65°C for 15 minutes, vortexed every 5-minutes and centrifuged at 15000 rpm at 4°C for 5 minutes. The aqueous top layer was transferred and purified with one volume of chloroform: isoamyl alcohol (24:1) and centrifuged for 10 minutes at 15000 rpm. The supernatant was mixed with 0.6 volume of cold isopropanol to precipitate the nucleic acid, mixed gently by inversion and centrifuged at 15000 rpm for 20 minutes. The precipitated pellet was washed with 100 uL of cold 70% ethanol and centrifuged for 5 minutes and air dried. RNA preparations were subjected to on-column DNase digestion (RNA clean and concentrator kit; ZymoResearch) and reverse- transcribed in the presence of random hexamers (LunaScript®RT SuperMix Kit, NEB *Inc*.) in 20 μl of total reaction volume containing 1 μg of total RNA and incubated at 55°C for 15 minutes following manufacturer’s instructions. Primer pairs used for qRT-PCR were designed for the three selected candidate genes (TK, GST and PIF3) with the support of the Integrated DNA Technologies’ PrimerQuest software (S2 Table). Three different housekeeping genes; Adenine phosphoribosyl transferase (APT), Beta-Tubulin (Tub) and TIP41-like family protein (TIP41), were tested. Based on the results of melt and standard curves, Beta-Tubulin was chosen as the endogenous reference for quantification of targets genes. RT-PCR reaction volumes were set up to 12.5 μL containing Luna Universal qPCR Master Mix (New England Biolabs *Inc*.), 2 μl of a 1:10 dilution of cDNA reaction, and 400 nM each of corresponding forward and reverse primers. Five different biological replicates represented by total RNA extracted from three individual plants and reverse transcribed into cDNA by LunaScript®RT SuperMix Kit (New England Biolabs *Inc*.) were used for statistical analysis of the quantification. Each cDNA sample was amplified in duplicate on a single 96-well optical plate using LightCycler480 (Roche). The cycling profile consisted of 95°C for 10 min followed by 40 cycles of 15 s at 95°C and 60 s at 60°C, as recommended by the manufacturer. Immediately after the final PCR cycle, a melting curve analysis was done to determine specificity of the reaction.

### Data analysis

For analysis of data on phenotypic traits, multiple correspondence analyses (MCA) was performed using FactoMineR package [26] in R software [27] to detect the association between sex types and morphological variables.

For SuperSAGE data analysis, sequence reads obtained from the different libraries were first sorted into different flowering groups based on their respective index sequences. Then, the subsequent extraction of SuperSAGE tags from reads was conducted using a script written in Perl. The R package edgeR [28] was used to determine differentially expressed tags between pairs of flowering groups (male vs female, male vs monoecious and female vs monoecious). Tags that were expressed in at least one sample or flowering group with a minimum number of 10 were considered for the analysis. Additionally, since two replicate samples were sequenced from each flowering group, tags that were represented in both replicates were considered. The expression cutoff was 100 counts per million (CPM), corresponding to a read count of about 5 for each tag.

For annotation of SuperSAGE tags, the selected tags were first aligned to the draft *D*. *rotundata* scaffold sequence, followed by extraction of the 2000-bp upstream sequences. These sequences were fused as queries for BLAST search against the non-redundant (nr) database of the National Center for Biotechnology Information (NCBI) and the Universal Protein Resource (UniProt).

For qRT-PCR analysis, quantities of RNA accumulation levels were calculated as relative quantification (RQ) values using the comparative cycle threshold (Ct) (2–ΔΔCt) method [29]. Before quantitative analyses, validation experiments were carried out to confirm equal amplification efficiencies between the reference and target genes and the applicability of the comparative method. Assessing the relative amplification efficiencies was achieved by running standard curves for each amplicon (i.e., five serial log10 dilutions of starting cDNA were amplified and Ct values of target and reference genes were measured in triplicate and plotted against the log of the input cDNA amount). The efficiencies were considered comparable when falling within a range of 100 ± 10%, corresponding to a curve slope of –3.3 ± 0.33 [30], and all primers pairs that did not allow PCR performances matching these criteria were discarded.

## Results

### Variation in flowering patterns and morphological traits in *D*. *rotundata* genebank accessions

**Fig 2:**
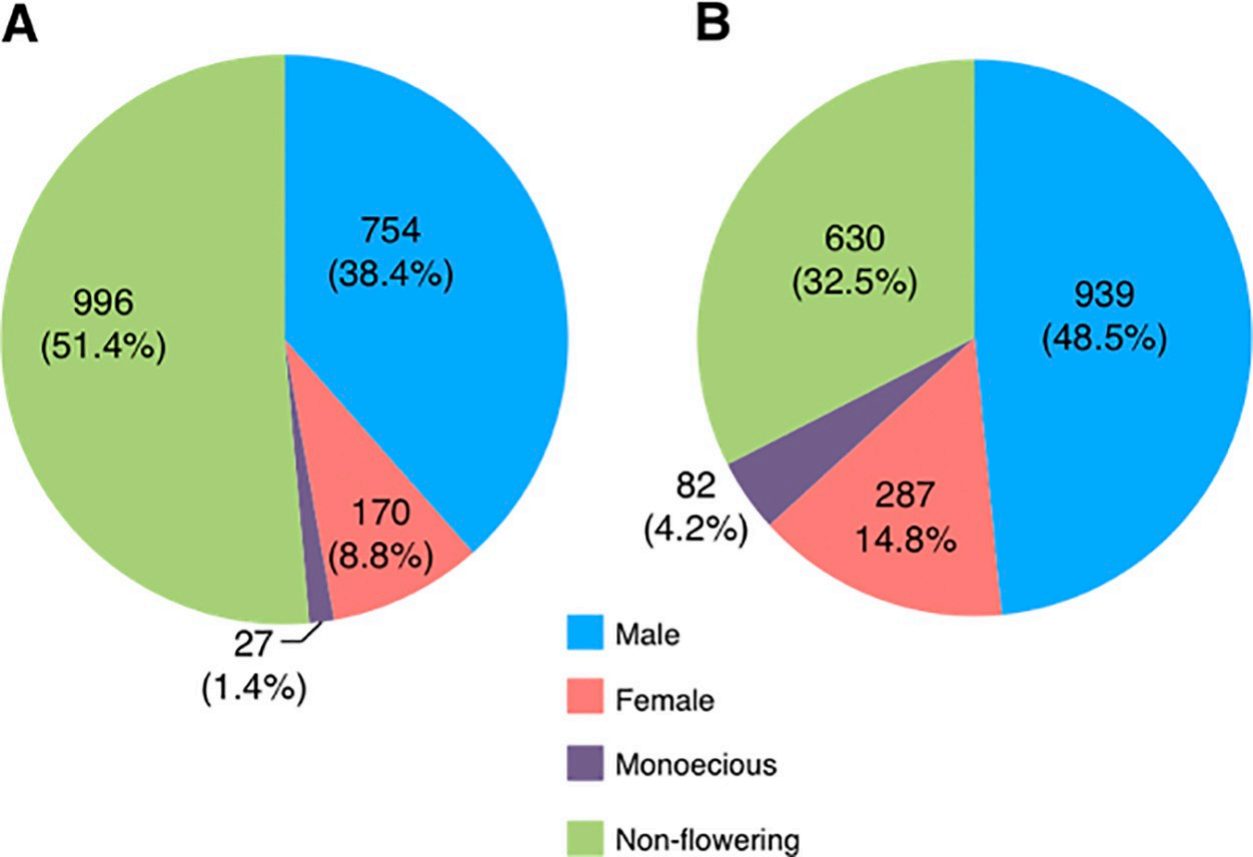
Sex distribution in yam (*D*. *rotundata*) accessions. The proportion of male, female, monoecious, and non-flowering accessions among 1938 genebank accessions in (A) 2010 and (B) 2011 growing seasons.

The flowering patterns of 1938 *D*. *rotundata* accessions collected primarily from the main yam growing regions of West and Central Africa were assessed under field conditions at IITA over two consecutive growing seasons in 2010 and 2011. *D*. *rotundata* accessions are easily distinguished based on morphology of their flowers as female, male, or monoecious (unisexual female and male flowers on the same plant) (Fig 1). In 2010, majority of the accessions (996, 51.4%) failed to flower, while 745 (38.4%), 170 (8.8%), and 27 (1.4%) were male, female, and monoecious, respectively (Fig 2). In the 2011 growing season, 939 (48.5%) accessions were male, followed by 630 (32.5%) non-flowering, 287 (14.8%) female, and 82 (4.2%) monoecious accessions (Fig 2B). Most accessions were consistent over the two seasons with respect to flowering, while some were not. About 326 of the male, 169 of the female and 43 of the monoecious accessions failed to flower at least in one of the seasons, hence the discrepancy observed between the two seasons with regard to the proportion of accessions with different sex groups (Fig 2). Overall, majority of *D*. *rotundata* accessions maintained at IITA were either male or non-flowering, while the female accessions represented less than 15% of the collection. Whereas, monoecious types are very rare in *D*. *rotundata*.

**Fig 3:**
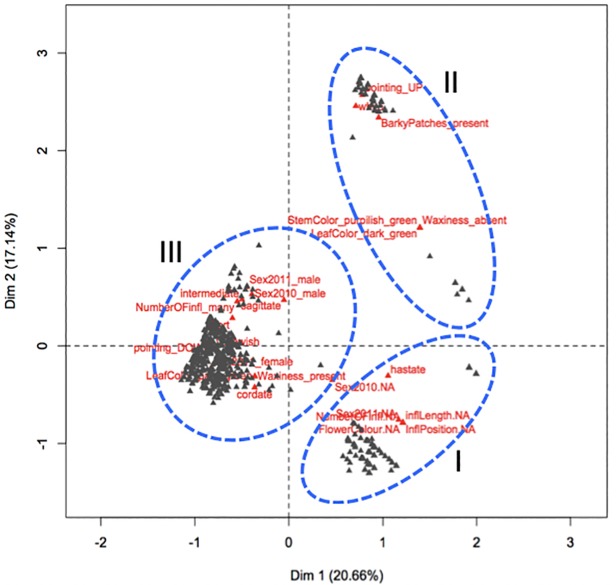
Multiple correspondence analysis (MCA) of sex type and phenotypic traits in yam (*D*. *rotundata*). The pattern of relationship between individual plants (black triangles) and the 20 most discriminant morphological traits (red triangles) are provided. The blue circles with broken lines represent the three main cluster: Cluster I = non-flowering accessions (hastate leaf shape, absence of flowering during 2010 and 2011 with no information on inflorescence length, shape position and color); Cluster II = male accessions (purplish green with barky patches and no waxiness on stem, dark green leaf, white and pointing up inflorescence); Cluster III = male, female, and monoecious accessions (presence of both male and female flowers during 2010 and 2011 with many, short to intermediate, pointing downward, yellowish inflorescence, dark green leaf with cordate and sagittate leaf shapes).

In addition to flowering and type of sex, we collected categorical data on 14 selected morphological traits over the same period (S1 Table). The data was subjected to multiple correspondence analysis (MCA), generating three major clusters that mainly reflected sex of the accessions (Fig 3). This suggested that certain morphological traits are associated with sex in *D*. *rotundata* with the two dimensions explaining about 38% of the total variations. Included in the first cluster were a group of non-flowering accessions that could be distinguished by traits such as purplish green stem, non-waxy stem, stem with non-barky patches, dark green leaf color, and hastate leaf shape. A second cluster composed entirely of male accessions was correlated with purplish green stem, presence of barky patches, non-waxy stem, dark green leaf, and sagittate leaf shape. The third group was composed of a mixture of male, female and monoecious flowering accessions that were identified by waxiness, absence of barky patches, either green, brownish green or purple stem color, and pale green or green leaf color as distinct traits.

### Generation and analysis of SuperSAGE tags

**Fig 4:**
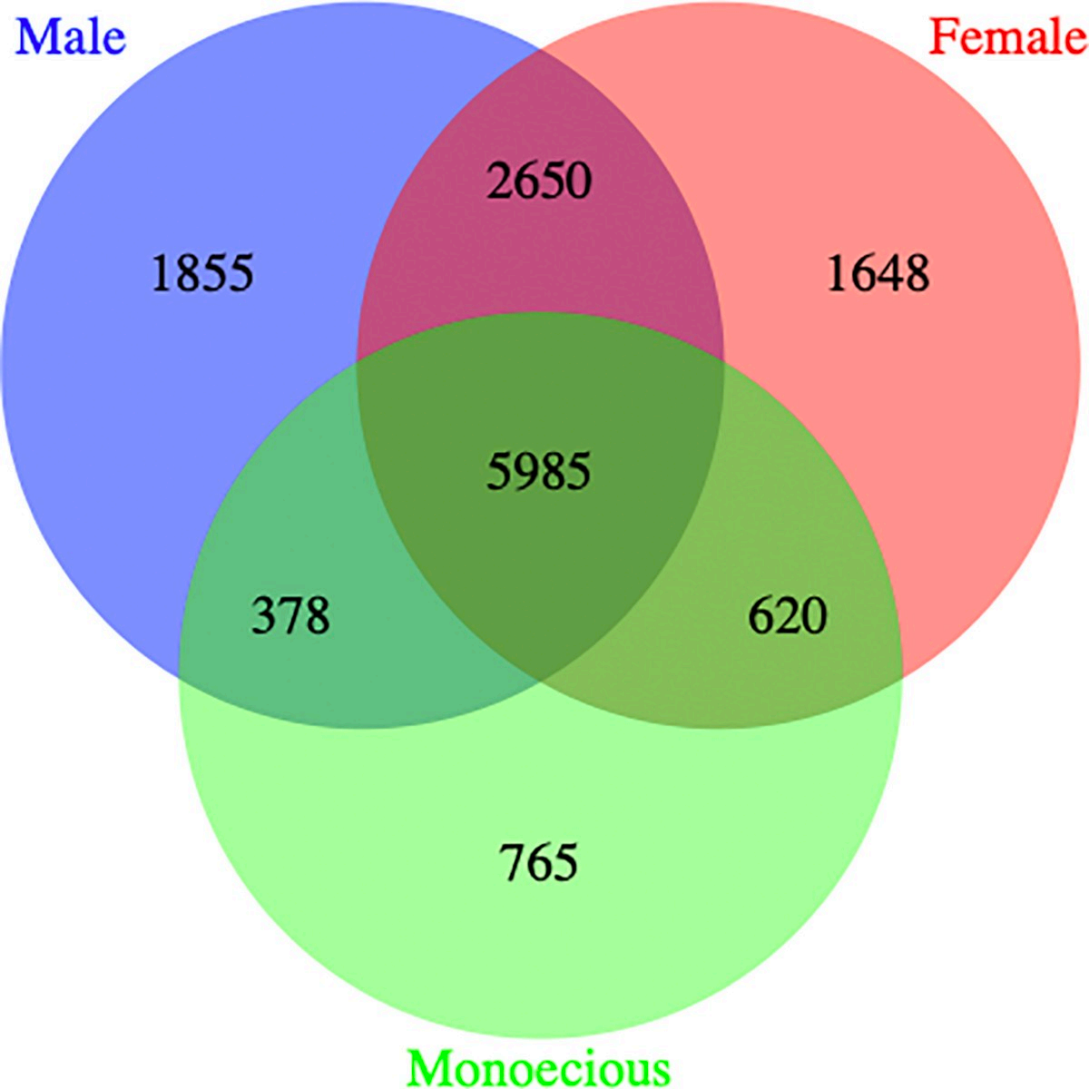
Distribution of SuperSAGE tags in flower buds of three yam (*D*. *rotundata*) sex types. The unique tags, as well as tags shared among male, female and monoecious plants were presented.

SuperSAGE libraries representing early stage male, female, and monoecious flower were multiplexed and sequenced on a single lane of an Illumina Genome Analyzer IIx, generating a total of 8,332,523 tags following quality control (QC) based on sequence read length, adapter dimmer, and sequencing error rates (Table 1). After the tags were sorted into the different flowering groups using Adapter1 sequences and singleton tags were removed, 20,236 unique non-singleton tags were obtained. Of these, 6,335 tags that were ten or less in number per library were removed, while the remaining 13,901 abundant tags were retained for further analysis (S3 Table). Of 13,901 abundant tags, 5985 (43%) were shared among the three flowering groups, whereas 1855 (13%), 1648 (12%) and 765 (5%) tags were specific to male, female, and monoecious flowers, respectively (Fig 4). The remaining tags were shared between male and female (2650 tags; 19.0%), male and monoecious (378 tags; 2.7%), and female and monoecious (620 tags; 4.5%) flower groups (Fig 4).

### Identification of genes differentially expressed across different flowering groups of yam

**Fig 5:**
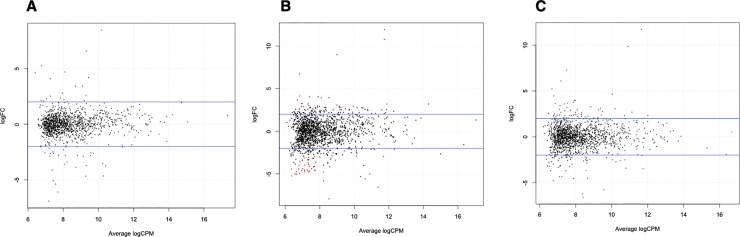
Differentially expressed genes in flower buds of three yam (*D*. *rotundata*) sex types. The abundance of genes differential expressed in (A) male and female, (B) male and monoecious, and (C) female and monoecious flowers buds are shown. Differentially expressed tags are represented by red dots. Fold change values between groups are plotted against average log expression values (standardized read counts) The logFC indicates the fold changes of differential expression whereas logCPM indicate count per million or tag/gene abundance. The horizontal blue lines represent 4-fold changes.

The fold change of differential expression and gene abundance (count per million) of 13,901 unique tags was compared across the different flowering groups. A total of 100 tags were differentially expressed with *p* and false discovery rate (FDR) values of less than 0.01 (Fig 5; S4 Table). The number of differentially expressed genes-DEGs (or SuperSAGE tags) between male vs. female, male vs. monoecious and female vs. monoecious groups were 13, 67 and 20, respectively (S4 Table). Of the 13 tags differentially expressed between male and female groups, five were highly expressed in male while the remaining eight were expressed in the female sex type. Similarly, the male vs monoecious group comparison revealed that 25 tags were highly represented in male, and 42 were abundant in monoecious sex type. For the female vs monoecious flower group, 11 and nine tags were expressed highly in female and monoecious flowers, respectively. The tag abundance estimated by the average logCPM (counts per million) ranged from a minimum of 6.36 to 11.88 for all the DEGs (Fig 5 and S4 Table).

### Annotation of SuperSAGE tags

Of the 100 differentially expressed tags, 88 tags were unique. These unique tags were aligned to the *D*. *rotundata* draft scaffold sequence to extract 2000-bp upstream sequences for use as a query for tag annotation by BLAST search against the National Center for Biotechnology Information (NCBI) and Universal Protein Resource (UniProt) databases. Of the 88-unique sequences, 87 could be aligned to the draft *D*. *rotundata* sequence, and 72 of these matched sequences available in the NCBI and UniProt databases. Sequences obtained for fifteen (17.24%) tags did not match sequences available in the two databases, while eight (9.09%) tags generated high e-values (>0.005). Of the 72 tags that could be annotated, 14 (19.5%) corresponded to proteins of unknown function, unnamed proteins, uncharacterized proteins or hypothetical proteins (S5 Table). However, a set of 18 (25.0%) tags representing 16 unique genes corresponded to genes that have been previously described as having a role in flower development and/or being expressed in flowers in multiple species (Table 2).

**Table 2:** Expression levels of superSAGE tags with respective candidate genes and biological roles in flower development across multiple species.

Tag sequence	M	F	Mo	Putative gene/protein	Species	Biological role or function	Source/ references
**A. Expressed in flowers**							
CATGAACTACGGCCCTGGTGCCGCCG	++	**±**	+	Pectinesterase inhibitor	*Lycoris aurea*	Unknown	Micheli et al 1998
CATGCATGCGTGGATGGGTGGACGTA	++	++	+	AQPs/MIP	*Oryza sativa Japonica* group	Unknown	Alexandersson et al, 2005
CATGCGTGGATGGGTGGACGTAGTTT	++	++	+	AQPs/MIP	*Oryza sativa Japonica* group	Unknown	Alexandersson et al, 2005
CATGGCTGAGGACGGCGAAGGTGGTG	++	**±**	+	Malonyltransferase	*Iris x hollandica*	Unknown	Suzuki et al, 2004
CATGCAGCCACTTGCCCTGTTTCCTT	+	**±**	++	VPE	*Gossypium arboreum*	Unknown	Alonso and Granell, 1995
CATGAAGATTGTCATTCCCTGAATTG	++	**±**	+	Trichome birefringence-like 23	*Theobroma cacao*	Unknown	Wang et al, 2008
**B. Transition from vegetative to reproductive phase**							
CATGTTGCTAGCTCAGCGGTTGGGTT	+	++	**±**	Long-chain fatty acid CoA synthetase	*Cucumis sativus*	Flower development	Smirnova et al, 2013
CATGTCCAAATGCCACTGGATATGTA	++	**±**	+	GDSL esterase/lipase APG	*Glycine soja*	Flower development	Ling 2008
**C. Flower color development**							
CATGCCAAGAAGTTTAGTGCTTGGAT	++	**±**	+	Glutathione-S transferase	*Hyacinthus orientalis*	Flower color intensity	Momose at al, 2013
**D. Flowering time, photoperiod and senescence regulation**							
CATGACTACATCTGGTCCTATGAATA	+	**±**	++	NAC domain protein	*Theobroma cacao*	Coordination of cold response and flowering time	Yoo et al, 2007
CATGCAAGTTCTACAGAGAATAAAAA	+	**±**	++	NAC domain protein	*Theobroma cacao*	Coordination of cold response and flowering time	Yoo et al, 2007
CATGATTAATTTGAAGACTGCTCAGT	+	**±**	++	Transferase family protein	*Populus trichocarpa*	Regulates flowering time	Wang et al, 2012
CATGCCGACGCCTTTGTCCACGCCAC	++	**±**	+	Zinc finger family protein	*Populus trichocarpa*	Regulates flowering time	Yang et al, 2014
CATGGGGATCCCCAATAGCATCTCCA	++	**±**	+	LOX	*Malus domestica*	Regulates flower senescence and flower opening	Liu and Han, 2010
CATGGGTGTCCCTTCCCAAAGGTAAG	**±**	++	+	RPS4	*Gossypium arboreum*	Regulates flowering time	Ai-Hua et al, 2014
CATGTGCGCTGCCTCAAATTTGCAAG	+	**±**	++	PIF3	*Medicago truncatula*	Regulates flowering time	Oda et al, 2004
**E. Sex specific expression**							
CATGTGACTTAACCGCAACCAGGGAA	+	**±**	++	DnaJ-like protein	*Glycine max*	Male flower development	Futamura et al, 1999
CATGCTCCGGCCCGCTGATGGGAATG	+	++	**±**	TK	Camellia sinensis	Female flower development	Bi et al, 2013

M, male; F, female; Mo, monoecious; ++ = high, + = medium, **±** = low expression.

Among the 16 genes reported to have involvement in flowering and flower development, twelve were highly expressed in female flowers, while the remaining two had very low expression levels in the same flowers. They genes also had variable expression levels in male and monoecious flowers (Table 2). Our results further indicated that 16, 15, and four genes were preferentially expressed in male, monoecious, and female flowers, respectively. However, only one gene was differentially expressed across all the flowering groups, whereas two, three and 14 genes were differentially expressed between female and monoecious, male and female, and male and monoecious flowers, respectively.

We conducted BLASTN search of the 88 deferentially expressed tags to *D*. *rotundata* scaffolds, eight tags returned multiple hits while 62 tags had a single hit each. The remaining 18 tags had no hit (S6 Table). The 62 tags with single hit were retained for further analysis. Further conversion of the scaffold position of the tags to recently published [9] pseudo chromosome as “TDr96_F1_Pseudo_Chromosome_v1.0.fasta”

The tags were distributed across all chromosomes except chromosome_01, with number of tags per chromosome ranging from 2 to 11 on chromosome_05 (S6 Table). Moreover, the gene ID and annotations of the tags were confirmed using the published [9] gene model, “TDr96_F1_v1.0. gff3”.

### Validation of selected candidate genes by qRT-PCR

To independently validate our SuperSAGE results using a different technique, we selected three representative genes associated with flowering and included Transketolase (TK), Glutathione-S Transferase (GST) and Phytochrome Interacting Factor-3 (PIF3). Three different housekeeping genes previously used for qRT-PCR across different organs and developmental stages of *Dioscorea opposita* [31] including Adenine Phosphoribosyl Transferase (APT), Beta-Tubulin (Tub) and TIP41-like family protein (TIP41) were tested based on standard curve and melt curve analysis.

**Fig 6:**
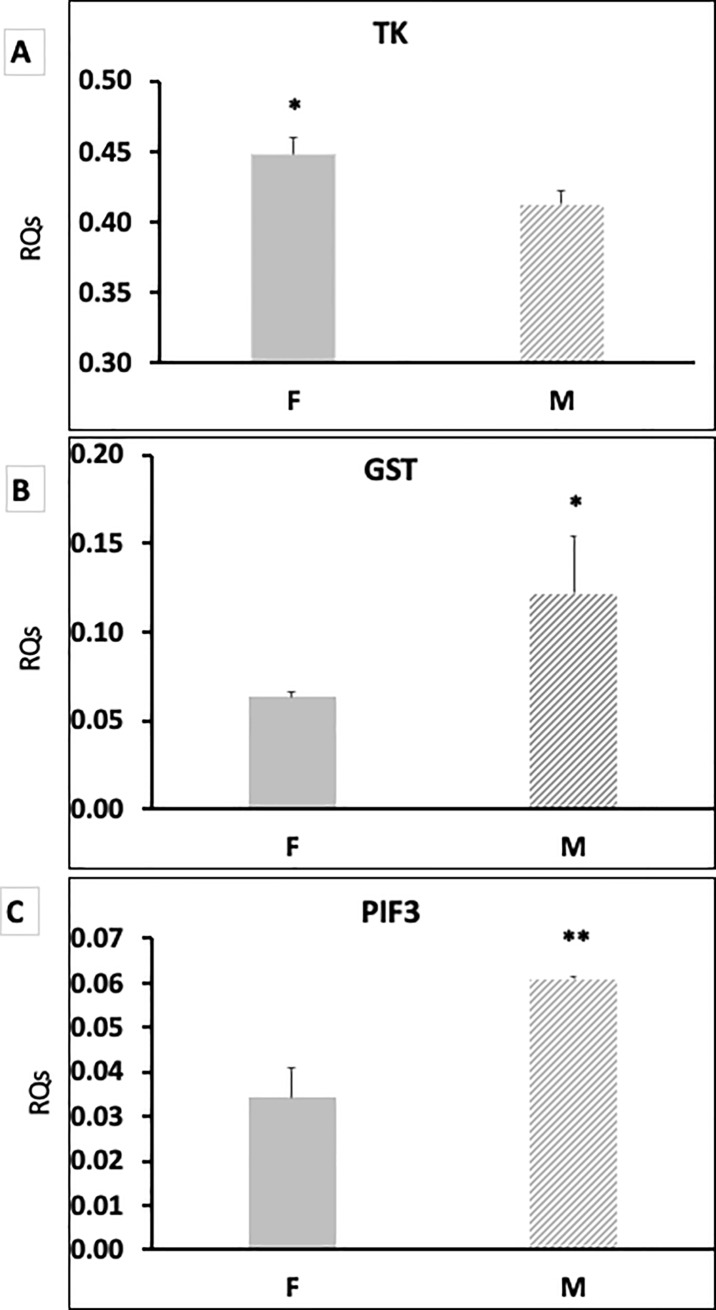
Overview of the qRT-PCR validation of selected differential expressed genes. Relative quantities (RQs) of (A) Transketolase (TK), (B) Glutathione S-transferase-like (GST), and (C) Phytochrome interacting factor-3 (PIF3) measured across female (F, solid) and male (M, pattern) sex types in *D*. *rotundata*. Error bars represent the standard error. Significance was determined by Student's t-test (* P<0.05, **P<0.01). Each value is the mean of five biological replicates.

Each target gene was amplified in two different samples; female (F) and male (M) flowers and in five biological replicates. Beta-Tubulin was selected in the current experiments as the most suitable and stable housekeeping gene in *D*. *rotundata*. qRT-PCR amplification revealed higher expression of TK in F than in M (Fig 6A) whereas, both GST and PIF3 were highly expressed in M compared to their level of expression in F (Fig 6B and 6C).

## Discussion

### Flowering relationships with morphological variation

A number of previous studies have shown the existence of dioecious and monoecious flowering patterns in *D*. *rotundata* [32–34]. Here, we have also identified dioecious and monoecious, as well as non-flowering accessions in the *D*. *rotundata* germplasm maintained at IITA. The monoecious plants predominantly produced male flowers, a phenomenon previously described as trimonoecious [32]. Most of the inflorescences consisted only of male flowers, while a limited number of inflorescences contained few female flowers.

Our observations over the two seasons demonstrate that majority of the *D*. *rotundata* accessions being maintained at IITA are either male or non-flowering types, with a significantly lower proportion of female accessions, while monoecious accessions are rare (Fig 2). Abundance of flowering appeared to vary according to sex, as male accessions flowered profusely compared to female ones. Moreover, higher numbers of male flowers develop on monoecious plants than female flowers. A similar observation from materials collected in Benin showed that female plants are rare and produce a limited number of flowers, whereas the male plants, whenever they flower, produce flowers in abundance [33]. Flowering pattern was largely inconsistent between the two seasons. This could be related to the effect of environmental factors as previously reported [32]. This supported by the differences in mean monthly temperature and rainfall we recorded at the IITA experimental site in 2010 and 2011, particularly during main flowering period between July and September (S7 Table).

Morphological characters that could be used to predict or distinguish the different flowering groups or sex types at the earliest growth stage possible are important particularly for breeders to select germplasm and cultivars in breeding experiments. Our study revealed that a set of selected morphological traits can be used to for predict sex in *D*. *rotundata* accessions (Fig 3; S1 Table). The correlation between flower type, morphological traits, and ploidy level previously reported in *D*. *rotundata* suggested that all triploid individuals are either male or non-flowering, and display some morphological features distinct from diploid individuals [35]. This morphological traits-based prediction of sex and ploidy level provides significant practical advantages for choosing of parents for crossing in breeding trials. However, we do recognize that the correlation identified needs to be validated with repeated experiments conducted under different environmental conditions to select those traits with the highest predictive value across multiple environments.

### Identification of candidate genes associated with flowering in yams

In our study, a number of genes differentially expressed in *D*. *rotundata* flower types were identified (Table 2 and S4 Table). However, only a few of the DEGs corresponded to genes known to be associated with flowering in plants, including those that are known either for their expression in flowers, for organ or sex specific expression, for a role in the regulation of flowering time, photoperiod and the transition from vegetative to reproductive phase, or for flower color development (Table 2). Examples of candidate genes with expression in flowers include the *Pectinesterase inhibitor* and *AQPs/MIP* gene in flowers of *Arabidopsis thaliana* [36,37] the *Malonyltransferase* gene in flowers of *Salvia splendens* [38], and the *VPE* gene in *Citrus sinensis* L. during flower development [39] and expression of *Trichome birefringence-like 23* at mature pollen stage [40] and in petal, sepal, pedicel, stamen, pollen, and petal differentiation and expansion stages of *Arabidopsis thaliana* [41,42].

In tomato, the long chain fatty acid CoA synthetase gene that is highly expressed in anther and petals, specifically in the sites subject to epidermal fusion [33], has been shown to be important for flower development, as lack of this gene impairs fertility and floral morphology. Similarly, the *GDSL esterase/lipase and APG* genes have been suggested for its potential involvement in flowering [43]. Among genes with a role in flower color development, our SuperSAGE analysis identified a *glutathione S-transferase-like* gene, reported for flower color intensity in *Dianthus caryophyllus* L [44].

Additional genes identified include those involved in flowering time, photoperiod and senescence regulation, such as: (1) a gene encoding NAC domain containing protein important for the coordination of cold response and flowering time [45], (2) a gene encoding a Transferase family *protein*, which is implicated in regulation of flowering time via the flowering repressor FLOWERING LOCUS C in *Arabidopsis thaliana* [46], a Zinc finger family *protein* gene involved in regulating flowering time and abiotic stress tolerance in *Chrysanthemum morifolium* [47], and a *LOX* gene known to regulate cell death related to flower senescence and flower opening [48,49]. A dramatic increase in *LOX* gene in response to senescence was observed in *Rosa hybrida* cv. Kardinal [42], in addition the *RPS4* gene is considered to play an important role in regulating flowering time, where a delay in flowering time was showed by silencing of the genes encoding *RPS4* and *Rhodanese* in *Glycine max* [50]. The transcription factor *PIF3* is considered to play an important role in the control of flowering through clock-independent regulation of CO and FT gene expression and is associated with early flowering in *Arabidopsis thaliana* [51]. Furthermore, genes known for organ specific expression were also identified including the DnaJ-like *protein* gene, detected predominantly in male flower of *Salix bakko* [52]. Likewise, transcriptome analysis identified male specific expression of the MYB-like gene *Male Specific Expression 1* (*MSE1*) in the dioicous species of *Asparagus officinalis* [53]. Unlike *D*. *rotundata*, (ZW) sex determination in *A*. *officinalis* follows the XY system and *MSE1* is tightly linked to the Y chromosome as well as specifically expressed in early stage of anther development.

Another gene identified by our SuperSAGE analysis was Transketolase (TK), whose over-expression was previously shown to result in a higher ratio of female flowers and increased yields in cucumber [54]. *In D*. *rotundata*, we found that *TK* is located on chromosome 11 (S6 Table). This corresponds to the same chromosomal region candidate where a candidate gene for sex determination was previously mapped by next-generation sequencing-based bulked segregant analysis (BSA) [9].

In this study, we have identified genes that are differentially expressed in *D*. *rotundata* male, female and monoecious flowers, including genes implicated for roles in flowering and flower development in multiple plant species. However, the mechanisms of flowering and sex determination in these species and the role these genes play remains largely unknown. Detailed functional characterization of the genes including analysis of allelic variants such as null and hypomorphic alleles, over and ectopic expression analyses etc can improve our knowledge of the underlying mechanisms. With the recent releases of a draft genome sequence of *D*. *rotundata* and a protocol for genetic transformation of the same species [9, 55], such studies will provide powerful tools for dissection of the molecular networks controlling sex determination and flowering in yams and for genetic improvement of the crop.

### Validation of selected candidate genes associated with flowering in yams

To validate the SuperSAGE data, we selected three of the DEGs and analyzed their expression levels in male and female flowers using qRT-PCR. Although samples for qRT-PCR analysis were obtained from plants independently grown from those used for SuperSAGE analysis, the results confirmed our SuperSAGE data for all three genes analyzed. Considering that one of the genes we identified and validated in this study, *TK*, is located within the same chromosomal region to which a candidate gene for sex determination in *D*. *rotundata* was previously mapped by NGS-based BSA [7], detailed functional characterization of these genes will improve how knowledge of how flowering and sex determination is regulated in yams.

## Conclusions

The current study is the first to identify candidate genes associated with flowering and sex determination in yams using a genome-wide transcriptome profiling approach. Once validated with further experiments, the candidate sex genes identified in this study can be targeted for manipulation of flowering. Such genes provide important inputs for studies aiming to overcome the erratic to non-flowering nature of yam, thereby contributing to improving breeding efficiency in the crop. This has a great practical significance considering most *D*. *rotundata* accessions are either non-flowering or flowering is inconsistent from year to year. In addition to the need for further genetic and genomic studies on flowering and sex determination in yam, understanding the environmental and epigenetic factors controlling sex and flowering regulation is invaluable to underpin the improvement of breeding systems for yam.

## Supporting information

S1 Table Phenotypic traits used for characterization of *D*. *rotundata* accessions. (DOCX)

S2 Table Details of primers and their sequences used for qRT-PCR analysis. (DOCX)

S3 Table Summary of all unique tags extracted from SuperSAGE library and used for analysis of differential expression. (XLSX)

S4 Table Summary of diffrentially expressed SuperSAGE tags across different sex types. (XLSX)

S5 Table List of differentially expressed tags, sequence BLAST result and expression or involvement of the genes in flower and flower development. (XLSX)

S6 Table Locations of the differentially expressed tags within the *D*. *rotundata* genome. (XLSX)

S7 Table Mean monthly temperature and rainfall during 2010 and 2011 growing seasons at IITA, Ibadan. (XLSX)

## References

[1] Scarcelli N, Barnaud A, Eiserhardt W, Treier UA, Seveno M, d’Anfray A, et al A set of 100 chloroplast DNA primer pairs to study population genetics and phylogeny in monocotyledons. PLoS ONE. 2011;6 10.1371/journal.pone.0019954 PMC3102674 21637837

[2] Mignouna H, Abang MAR. Yams In: C K, editor. Genome mapping and molecular breeding in plants. vol. 3 Heidelberg, Berlin, New York, Tokyo: Springer; 2007 p. 271–96.

[3] Terauchi R KG. Sex determination in Dioscorea tokoro, a wild yam species In: C A, editor. Sex Determination in Plants. Oxford OX4 1RE, UK: BIOS; 1999.

[4] Lebot V. Tropical root and tuber crops: cassava, sweet potato,yams and aroids Wallingford, UK: CABI Publishers; 2009.

[5] Aryal R, Ming R. Sex determination in flowering plants: Papaya as a model system. Plant Science. 2014; 217–218: 56–62. 10.1016/j.plantsci.2013.10.018 24467896

[6] Ming R, Bendahmane A, Renner SS. Sex Chromosomes in Land Plants. Annual Review of Plant Biology. 2011; 62:485–514. 10.1146/annurev-arplant-042110-103914 21526970

[7] Martin F, Sex Ratio and Sex Determination in Dioscorea. Journal of Heredity 1966; 57(3):95–99

[8] Cormier F, Lawac F, Maledon E, Gravillon M-C, Nudol E, Mournet P, et al A reference high-density genetic map of greater yam (Dioscorea alata L.). Theoretical and Applied Genetics. 2019; 132(6): 1733–1744. 10.1007/s00122-019-03311-6) 30783744 PMC6531416

[9] Tamiru M, Natsume S, Takagi H, White B, Yaegashi H, Shimizu M, et al Genome sequencing of the staple food crop white Guinea yam enables the development of a molecular marker for sex determination. BMC Biology; 2017; 15:86 10.1186/s12915-017-0419-x 28927400 PMC5604175

[10] Simpson GG, Dean C. Arabidopsis, the Rosetta stone of flowering time? Science. 2002 p. 285–9. 10.1126/science.296.5566.285 11951029

[11] Ma H, DePamphilis C. The ABCs of floral evolution. Cell. 2000; 101:5–8. 10.1016/S0092-8674(00)80618-2 10778850

[12] Coen ES, Meyerowitz EM. The war of the whorls: genetic interactions controlling flower development. Nature. 1991; 353:31–7. 10.1038/353031a0 1715520

[13] Heijmans K, Morel P, Vandenbussche M. MADS-box genes and floral development: The dark side. Journal of Experimental Botany. 2012; 63(15):5397–404. 10.1093/jxb/ers233 22915743

[14] Su CL, Chen WC, Lee AY, Chen CY, Chang YCA, Chao YT, et al A modified ABCDE model of flowering in orchids based on gene expression profiling studies of the moth orchid Phalaenopsis aphrodite. PLoS ONE. 2013; 8(11): e80462 10.1371/journal.pone.0080462 24265826 PMC3827201

[15] Spigler RB, Lewers KS, Main DS, Ashman TL. Genetic mapping of sex determination in a wild strawberry, Fragaria virginiana, reveals earliest form of sex chromosome. Heredity. 2008; 101:507–17. 10.1038/hdy.2008.100 18797475

[16] Jaligot E, Adler S, Debladis É, Beulé T, Richaud F, Ilbert P, et al Epigenetic imbalance and the floral developmental abnormality of the in vitro-regenerated oil palm Elaeis guineensis. Annals of Botany. 2011; 108(8): 1453–62. 10.1093/aob/mcq266 21224269 PMC3219487

[17] Acosta IF, Laparra H, Romero SP, Schmelz E, Hamberg M, Mottinger JP, et al tasselseed1 is a lipoxygenase affecting jasmonic acid signaling in sex determination of maize. Science. 2009; 323:262–5. 10.1126/science.1164645 19131630

[18] Velculescu VE, Zhang L, Vogelstein B, Kinzler KW. Serial Analysis of Gene Expression. Science. 1995; 270:484–7. 10.1126/science.270.5235.484 7570003

[19] Saha S, Sparks AB, Rago C, Akmaev V, Wang CJ, Vogelstein B, et al Using the transcriptome to annotate the genome. Nature Biotechnology. 2002; 20:508–12. 10.1038/nbt0502-508 11981567

[20] Gowda M, Jantasuriyarat C, Dean RA WG. Robust-LongSAGE (RL-SAGE): a substantially improved LongSAGE method for gene discovery and transcriptome analysis. Plant Physiology. 2004; 134:890–7. 10.1104/pp.103.034496 15020752 PMC389912

[21] Nielsen KL, Høgh AL, Emmersen J. DeepSAGE—Digital transcriptomics with high sensitivity, simple experimental protocol and multiplexing of samples. Nucleic Acids Research. 2006; 34(19): e133 10.1093/nar/gkl714 17028099 PMC1636492

[22] Marioni JC, Mason CE, Mane SM, Stephens M, Gilad Y. RNA-seq: An assessment of technical reproducibility and comparison with gene expression arrays. Genome Research. 2008; 18:1509–17. 10.1101/gr.079558.108 18550803 PMC2527709

[23] Matsumura H, Yoshida K, Luo S, Kimura E, Fujibe T, Albertyn Z, et al High-throughput superSAGE for digital gene expression analysis of multiple samples using next generation sequencing. PLoS ONE. 2010; 5(8):e12010 10.1371/journal.pone.0012010 20700453 PMC2917361

[24] IPGRI/IITA. Descriptors for yam (Dioscorea spp.). Interna- tional Institute of Tropical Agriculture, Ibadan, Nigeria/International Plant Genetic Resources Institute Rome, Italy; 1997.

[25] Dellaporta SL, Wood J, Hicks JB. A plant DNA minipreparation: Version II. Plant Molecular Biology Reporter.1983; 1(4):19–21. 10.1007/BF02712670

[26] Lê S, Josse J, Husson F. FactoMineR: An R Package for Multivariate Analysis. Journal of Statistical Software. 2008; 25:253–8. http://hdl.handle.net/10.18637/jss.v025.i01

[27] R Development Core Team. R: A language and environment for statistical computing R Foundation for Statistical Computing, Vienna, Austria URL http://www.R-project.org/. R Foundation for Statistical Computing, Vienna, Austria. 2013.

[28] Robinson MD, McCarthy DJ, Smyth GK. edgeR: a Bioconductor package for differential expression analysis of digital gene expression data. Bioinformatics (Oxford, England). 2010; 26:139–40. 10.1093/bioinformatics/btp616 19910308 PMC2796818

[29] Livak KJ, Schmittgen TD. Analysis of Relative Gene Expression Data Using RealTime Quantitative PCR and the 22DDCT Method. Methods. 2001; 25, 402–408. 10.1006/meth.2001.1262 11846609

[30] Livak K. ABI Prism 7700 sequence detection system, User Bulletin 2. PE Applied Biosystems, Foster City, CA, U.S.A; 1997.

[31] Zhao X, Zhang X, Guo X, Li S, Han L, Song Z, Wang Y, Li J. Identification and Validation of Reference Genes for qRT-PCR Studies of Gene Expression in Dioscorea opposita. BioMed Research International. 2016; 2016(1):1–13. 10.1155/2016/3089584 PMC4899605 27314014

[32] Hamadina EI, Craufurd PQ, Asiedu R. Flowering intensity in white yam (Dioscorea rotundata). Journal of Agricultural Science. 2009; 147:469–77. 10.1017/S0021859609008697

[33] Dansi A, Mignouna HD, Zoundjihékpon J, Sangare A, Asiedu R, Quin FM. Morphological diversity, cultivar groups and possible descent in the cultivated yams (Dioscorea cayenensis/D. rotundata) complex in Benin Republic. Genetic Resources and Crop Evolution. 1999; 46:371–88. 10.1023/A:100869812

[34] Hamon P, Toure B. Characterization of traditional yam varieties belonging to the Dioscorea cayenensis-rotundata complex by their isozymic patterns. Euphytica. 1990; 46:101–7. 10.1007/BF00022303

[35] Girma G, Hyma KE, Asiedu R, Mitchell SE, Gedil M, Spillane C. Next-generation sequencing based genotyping, cytometry and phenotyping for understanding diversity and evolution of Guinea yams. Theoretical and Applied Genetics. 2014; 127:1783–94. 10.1007/s00122-014-2339-2 24981608

[36] Micheli F, Holliger C, Goldberg R, Richard L. Characterization of the pectin methylesterase-like gene AtPME3: A new member of a gene family comprising at least 12 genes in Arabidopsis thaliana. Gene. 1998; 220:13–20. 10.1016/s0378-1119(98)00431-4 9767082

[37] Alexandersson E, Fraysse L, Sjövall-Larsen S, Gustavsson S, Fellert M, Karlsson M, et al Whole gene family expression and drought stress regulation of aquaporins. Plant Molecular Biology. 2005; 59:469–84. 10.1007/s11103-005-0352-1 16235111

[38] Suzuki H, Sawada S, Watanabe K, Nagae S, Yamaguchi MA, Nakayama T, et al Identification and characterization of a novel anthocyanin malonyltransferase from scarlet sage (Salvia splendens) flowers: An enzyme that is phylogenetically separated from other anthocyanin acyltransferases. Plant Journal. 2004; 38:994–1003. 10.1111/j.1365-313X.2004.02101.x 15165190

[39] Alonso JM, Granell A. A putative vacuolar processing protease is regulated by ethylene and also during fruit ripening in Citrus fruit. Plant Physiology. 1995; 109:541–7. 10.1104/pp.109.2.541 7480346 PMC157618

[40] Wang Y, Zhang W-Z, Song L-F, Zou J-J, Su Z, Wu W-H. Transcriptome analyses show changes in gene expression to accompany pollen germination and tube growth in Arabidopsis. Plant Physiology. 2008; 148:1201–11. 10.1104/pp.108.126375 18775970 PMC2577266

[41] Schmid M, Davison TS, Henz SR, Pape UJ, Demar M, Vingron M, et al A gene expression map of Arabidopsis thaliana development. Nature Genetics. 2005; 37:501–6. 10.1038/ng1543 15806101

[42] Smirnova A, Leide J, Riederer M. Deficiency in a Very-Long-Chain Fatty Acid -Ketoacyl-Coenzyme A Synthase of Tomato Impairs Microgametogenesis and Causes Floral Organ Fusion. Plant Physiology. 2013; 161:196–209. 10.1104/pp.112.206656 23144186 PMC3532251

[43] Ling H. Sequence analysis of GDSL lipase gene family in Arabidopsis thaliana. Pakistan Journal of Biological Sciences. 2008; 11:763–7. 10.3923/pjbs.2008.763.767 18819574

[44] Momose M, Itoh Y, Umemoto N, Nakayama M, Ozeki Y. Reverted glutathione S-transferase-like genes that influence flower color intensity of carnation (Dianthus caryophyllus L.) originated from excision of a transposable element. Breeding Science. 2013; 63:435–40. 10.1270/jsbbs.63.435 24399917 PMC3859356

[45] Yoo SY, Kim Y, Kim SY, Lee JS, Ahn JH. Control of flowering time and cold response by a NAC-domain protein in Arabidopsis. PLoS ONE. 2007;2(7): e642 10.1371/journal.pone.0000642 17653269 PMC1920552

[46] Wang M, Yan J, Zhao J, Song W, Zhang X, Xiao Y, et al Genome-wide association study (GWAS) of resistance to head smut in maize. Plant Science. 2012; 196:125–31. 10.1016/j.plantsci.2012.08.004 23017907

[47] Yang Y, Ma C, Xu Y, Wei Q, Imtiaz M, Lan H, et al A Zinc Finger Protein Regulates Flowering Time and Abiotic Stress Tolerance in Chrysanthemum by Modulating Gibberellin Biosynthesis. The Plant Cell. 2014; 26:2038–54. 10.1105/tpc.114.124867 24858937 PMC4079367

[48] Liu S, Han B. Differential expression pattern of an acidic 9/13-lipoxygenase in flower opening and senescence and in leaf response to phloem feeders in the tea plant. BMC Plant Biology. 2010; 10:228 10.1186/1471-2229-10-228 20969806 PMC3095316

[49] Fukuchi-Mizutani M, Ishiguro K, Nakayama T, Utsunomiya Y, Tanaka Y, Kusumi T, et al Molecular and functional characterization of a rose lipoxygenase cDNA related to flower senescence. Plant Science. 2000; 160:129–37. 10.1016/S0168-9452(00)00373-3 11164585

[50] Ai-Hua S, Yin-Hua C, Zhi-Hui S, Xiao-Juan Z, Xue-Jun W, De-Zheng Q, et al Identification of photoperiod-regulated gene in soybean and functional analysis in Nicotiana benthamiana. Journal of Genetics. 2014; 93:43–51. 10.1007/s12041-014-0331-x 24840822

[51] Oda A, Fujiwara S, Kamada H, Coupland G, Mizoguchi T. Antisense suppression of the Arabidopsis PIF3 gene does not affect circadian rhythms but causes early flowering and increases FT expression. FEBS Letters. 2004; 557:259–64. 10.1016/s0014-5793(03)01470-4 14741378

[52] Futamura N, Ishiiminami N, Hayashida N, Shinohara K. Expression of DnaJ homologs and Hsp70 in the Japanese willow (Salix gilgiana Seemen). Plant and Cell Physiology. 1999; 40:524–31. 10.1093/oxfordjournals.pcp.a029573 10427775

[53] Murase K, Shigenobu S, Fujii S, Ueda K, Murata T, Sakamoto A, Wada Y, Yamaguchi K, Osakabe Y, Osakabe K, Kanno A, Ozaki Y, Takayama S. MYB transcription factor gene involved in sex determination in Asparagus officinalis. Genes Cells. 2017; 22(1):115–123. 10.1111/gtc.12453 27869347

[54] Bi H, Wang M, Dong X, Ai X. Cloning and expression analysis of transketolase gene in Cucumis sativus L. Plant Physiology and Biochemistry. 2013; 70:512–21. 10.1016/j.plaphy.2013.06.017 23860231

[55] Nyaboga E, Tripathi JN, Manoharan R, Tripathi L. Agrobacterium-mediated genetic transformation of yam (Dioscorea rotundata): an important tool for functional study of genes and crop improvement. Fronteirs in Plant Science. 2014; 5:463 10.3389/fpls.2014.00463 25309562 PMC4164099

