# Supplementary material for: Identification of candidate flowering and sex genes in white Guinea yam (D. rotundata Poir.) by SuperSAGE transcriptome profiling
Source: PLoS One. 2019 Sep 23;14(9):e0216912. doi: 10.1371/journal.pone.0216912 (PMC6756524; doi:10.1371/journal.pone.0216912)
Supplement: S1 Table — (DOCX) [file pone.0216912.s001.docx]

**Table S1 Phenotypic traits used for characterization of D. rotundata accessions.**

| **Morphological descriptors** | **Parameters used** |
| --- | --- |
| Sex | 1=female, 2=male, 3=monoecious and 4= no flowering |
| Inflorescence position | 1=pointing upward and 2= pointing downward |
| Average length of inflorescence | ≤5cm=short, 6-15=intermediate and ≥ 16cm=long |
| Number of inflorescence per plant | <10=few, 11-29=medium and ≥ 30=many |
| Number of inflorescence per internode | Count |
| Flower color | 0=not available, 1=white and 2=yellowish |
| Stem color | 1=green, 2=brownish green, 3=purplish green and 4=purple |
| Vigour | 3=low, 5= intermediate and 7=high |
| Leaf color | 1=green, 2=yellowish green and 3=dark green |
| Leaf shape | 1=hastate, 2=sagitate and 3=cordate |
| Presence of barky patches on stem | 0=absent and 1=present |
| Presence of waxiness on stem | 0=absent and 1=present |
