# Supplementary material for: Identification of candidate flowering and sex genes in white Guinea yam (D. rotundata Poir.) by SuperSAGE transcriptome profiling
Source: PLoS One. 2019 Sep 23;14(9):e0216912. doi: 10.1371/journal.pone.0216912 (PMC6756524; doi:10.1371/journal.pone.0216912)
Supplement: S2 Table — (DOCX) [file pone.0216912.s002.docx]

**Table S2.** Details of primers and their sequences used for qRT-PCR analysis

| **Name** | **Sequence** |
| --- | --- |
| DrGST-qPCR_F | TGTGGTGAGAGTCAGGATAG |
| DrGST-qPCR_R | TGTAAACAGGGTTGGACTTG |
| DrPIF3-qPCR_F | AGGGTAGTGATAGGTTGAGG |
| DrPIF3-qPCR_R | TTGGACCACTTATGCTCTTG |
| DrTK-qPCR_F | GAGGGTTGTCTCACTTGTATC |
| DrTK-qPCR_R | CACCAGCCTCAATACTAACTC |
| DrTub-qPCR_F | GACATCGAGCACAGAATCAA |
| DrTub-qPCR_R | CCAGATCTTTAGGCCTGATAAC |
| DrAPT-qPCR_F | GGTACTGACTGTTTGGAGATG |
| DrAPT-qPCR_R | TTACTGCAGCACGAAGTG |
| DrTIP41-qPCR_F | ACCACCTCATCATAGAAGAGTA |
| DrTIP41-qPCR_R | CCAAAGGAAAGTGTGGAAGA |
